# Immune Responses and Lassa Virus Infection 

**DOI:** 10.3390/v4112766

**Published:** 2012-11-05

**Authors:** Marion Russier, Delphine Pannetier, Sylvain Baize

**Affiliations:** 1 Unité de Biologie des Infections Virales Emergentes, Institut Pasteur, 21 avenue Tony Garnier, 69365 Lyon, France; Email: marion.russier@inserm.fr; 2 Laboratoire P4 Jean Mérieux-Inserm, 21 avenue Tony Garnier, 69365 Lyon, France; Email: delphine.pannetier@inserm.fr

**Keywords:** Lassa virus, hemorrhagic fever, T-cell responses, antigen-presenting cells, immunity

## Abstract

Lassa fever is a hemorrhagic fever endemic to West Africa and caused by Lassa virus, an Old World arenavirus. It may be fatal, but most patients recover from acute disease and some experience asymptomatic infection. The immune mechanisms associated with these different outcomes have not yet been fully elucidated, but considerable progress has recently been made, through the use of *in vitro* human models and nonhuman primates, the only relevant animal model that mimics the pathophysiology and immune responses induced in patients. We discuss here the roles of the various components of the innate and adaptive immune systems in Lassa virus infection and in the control of viral replication and pathogenesis.

## 1. Introduction

Lassa fever (LF) is a viral hemorrhagic fever (VHF) caused by Lassa virus (LASV), an Old World arenavirus [1]. LASV is an enveloped virus with two single-stranded RNA segments. The large segment encodes a small zinc-binding (Z) protein that plays a crucial role in the regulation of transcription and replication [2,3,4] and in viral budding [5,6,7], and the RNA polymerase (L). The small segment encodes the nucleoprotein (NP) and the two envelope glycoproteins (GP_1_ and GP_2_) mediating cell entry after binding to α-dystroglycan [8,9]. The disease is endemic in West Africa, including Nigeria, Liberia, Sierra Leone and Guinea, in particular, but LASV probably also circulates in neighboring countries such as Mali, Ivory Coast, Ghana, or Burkina Faso [10,11]. The attack rate of LF is difficult to be precisely quantified because of limited survey in endemic countries, similarity of clinical signs with more common diseases and elevated incidence of asymptomatic LASV infection. However, it is usually admitted that LASV may be responsible for about 300,000 cases and 5,000–6,000 deaths each year [12,13]. Humans become infected through contact with *Mastomys natalensis*, a peridomestic rodent, which acts as the reservoir host [13]. Large numbers of these rodents live in the vicinity of, and even within, residences, and 80% of the rodent population is infected with the virus. Contact between humans and infected reservoir animals is thus frequent in villages, and the seroprevalence of humans living in endemic zones may be as high as 50%. The disease is then transmitted between humans. Its severity ranges from asymptomatic infection to fatal HF, and nosocomial outbreaks are frequently observed [14]. Nonspecific signs, such as fever, headache, arthralgia, myalgia and severe asthenia, are observed six to 12 days after infectious contact. Pharyngitis, conjunctivitis, cough, abdominal pain, diarrhea and vomiting appear in the next few days. Cervical and facial edema, hemorrhages, renal and liver failures and, in some of the more severe cases, encephalopathy may occur. Death follows hypotensive, hypovolemic and hypoxic shock in severely affected patients, whereas the symptoms disappear 10 to 15 days after disease onset in surviving patients [15]. LF is a major public health and economic problem in the regions in which it is endemic, not only due to the limited health structures of these regions and the isolation of the affected populations, but mostly due to the high morbidity and disabling aftereffects, such as deafness, which occurs in one-third of all survivors and may persist throughout life [16]. There is currently no licensed vaccine against LF, and the only treatment available is based on ribavirin [17]. However, this molecule is not readily available in the countries in which LF is endemic, due to its high cost, and it must be administered early if it is to be effective. This drug is therefore far from satisfactory as a treatment for LF.

The observation that most patients successfully control LASV infection and recover, and that many cases of asymptomatic LASV infection do occur, suggests that LF can induce effective immunity. By contrast, severe LASV infection seems to be associated with defective immune responses and even immunosuppression [15,18]. The pathogenesis of LF and the immune responses occurring during the disease have yet to be fully elucidated. This limited knowledge results principally from the remote location of the areas in which LF is endemic and the high level of infectivity of LV, both of which have hampered investigations of LF in humans. In addition, there is no rodent model other than strain 13 guinea pigs, which can be infected but do not reproduce the pathophysiology or immune responses observed in humans [19]. Thus, nonhuman primates (NHP) represent currently the most relevant model for LF, but investigations in these animals are limited by the need to manipulate them in BSL4 facilities. Despite these problems, substantial advances have recently been made through studies in NHP and human *in vitro *models and the use of reverse genetic tools. We review these data here, providing an overview of current knowledge concerning the immune responses associated with LASV infection.

## 2. Pathogenesis of Lassa Fever

Antigen-presenting cells (APC)—dendritic cells (DC) and macrophages (MP)—are probably the first cells targeted by LASV [20,21]. The widespread distribution of these cells in the mucosal tissues and skin, results in their early infection, allowing the first replicative cycles to occur. APC are probably also responsible for spreading the virus and establishing systemic infection, due to their mobility and their presence in many organs and tissues [22]. Massive viral release then occurs in the secondary lymphoid organs and liver, and hepatocytes, fibroblasts, endothelial cells and some epithelial cells become targets for viral replication. However, changes in the endothelium and other organs do not seem to be severe enough to account for terminal shock and death, which seem instead to be linked to the host response. The most frequent microscopic alterations reported in patients and in NHP are multifocal hepatocellular necrosis with weak inflammatory cell involvement, adrenal cortical cell necrosis, substantial infiltration with mononuclear cells, mostly MP, in most organs, interstitial pneumonitis, acute myocarditis and damage to reticuloendothelial tissues [23,24,25,26,27]. Lymphadenopathy and splenomegaly are observed, and changes to lymphoid organs include the disruption of follicular architecture and the depletion of cells from the bone marrow, spleen and lymph nodes.

Transient lymphopenia affecting CD4^+^ and CD8^+^ T cells, NK cells and B cells is observed early in the disease, followed two weeks after disease onset by leukocytosis, principally involving neutrophils [18,22,25,26,28]. Moderate and transient thrombocytopenia is also a feature of LF, accompanied by a progressive depression of platelet function [18,25,28,29]. However, no important change in coagulation occurs, and disseminated intravascular coagulation is never observed during LF. Together, these changes are not severe enough to account for the hemorrhagic signs and plasma leakage observed in LF, which seem instead to be mostly due to an increase in endothelial permeability, probably induced by host factors. Plasma AST and ALT levels increase strongly in the terminal stages of the disease in both humans and NHP, whereas they increase in a transient and moderate manner in nonfatal cases of LF [17,22,25,26,30,31,32,33]. These events may reflect hepatic abnormalities. However, the high AST/ALT ratio suggests that the source of AST may be an organ other than the liver. Similarly, high concentrations of IL-6 have been detected in plasma during fatal LASV infections in NHP [22,25]. IL-6 production may be associated with hepatic regeneration during LF, a phenomenon described in NHP and humans [26,34], but it may also result from tissue damage in other organs and muscles. IL-6 may be involved in neutrophilia [35,36], and the simultaneous increases in AST levels and the number of circulating neutrophils in NHP suggest that tissue damage may result, at least in part, from neutrophil infiltration.

Severe LF is not the result of a single organ failure. Instead, it is associated with multiple-organ dysfunction, with death ultimately occurring in a context of hypoxic, hypovolemic and hypotensive shock. However, further investigations are required to elucidate more fully the pathogenic mechanisms ultimately leading to catastrophic illness and fatal infection.

## 3. Antigen-Presenting Cells Play a Key Role in Lassa Fever

Antigen-presenting cells (DC and MP) play a crucial role in the induction and regulation of immune responses. On the one hand, they are the key actors in innate immunity, mediating the induction of inflammatory responses and the direct control of viral infections. On the other hand, the ability of these cells to present antigens (Ag), enables DC and MP to initiate and to orchestrate adaptive humoral and cellular immune responses [37,38]. DC and MP are the primary target for LASV replication. APC are initially the site of early replication in the periphery, and, following the infection of most of these cells and the relentless replication occurring in the lymphoid organs, these cells become the primary reservoir for the systemic dissemination of LASV [22,25,29,31]. However, this dual role of APC is not without consequences for the immune responses induced during LF.

### 3.1. LASV Infection of APC

The privileged tropism of LASV for DC and MP has been demonstrated both *in vitro* in human cells and *in vivo* in NHP. Human DC and MP are highly permissive to LASV infection, leading to the release of large numbers of viral particles, particularly in DC, with no effect on cell viability [1,26,39]. LASV has recently been reported to bind plasmacytoid DC (pDC), suggesting that these cells are probably a viral target *in vivo*, as reported for lymphocytic choriomeningitis virus (LCMV) [40]. In NHP, most of the DC and MP are infected by day 7 in all lymph nodes, the splenic marginal zone and, to a lesser extent, in the red pulp, thymus and liver (Kupffer cells) [22], and the viral load in the lymphoid organs and liver remains high throughout the disease [22,25,30,31]. LASV infection induces no change in the viability of APC, resulting, at least *in vitro,* in the sustained release of large numbers of viral particles [20]. Mopeia virus (MOPV) is an Old World arenavirus closely related to LASV. Its amino acid sequences are about 75% identical to those of LASV and it shares the same rodent host, but is nonpathogenic in humans and NHP, and can even induce cross-reactivity and immune protection against subsequent LASV challenge [23,41,42,43]. This virus is thus studied in comparison with LASV, as a model of nonfatal LF or asymptomatic LASV infection. Like LASV, MOPV can infect DC and MP, resulting in the release of large numbers of viral particles with no apparent cytotoxicity [39,44]. These observations suggest that this privileged tropism of LASV for APC is not correlated with its high level of pathogenicity, being instead a common feature of arenavirus infections.

### 3.2. Responses of APC to LASV Infection and Correlation with Pathogenicity

APC are not activated by LASV infection. Despite the massive release of viral particles, LASV-infected DC do not produce inflammatory cytokines or express activation molecules at their surface [20,21,45]. Moreover, LASV infection does not lead to DC maturation [20]. This lack of DC activation and maturation in response to LASV infection may be associated with the immunosuppression observed in severe infection. Indeed, Ag presentation by immature DC is known to result in tolerance and defective immunity [46,47], and proinflammatory cytokines are crucial for the induction of adaptive immunity [48]. Furthermore, this lack of DC activation probably favors LASV replication, as immature DC produce significantly more LASV particles than mature DC [20]. Similarly, no significant activation is observed after the infection of MP with LASV, other than the production of small amounts of type I IFN [20,39,45]. This absence of MP activation may also favor viral spread, as MP activation is known to increase the microbicidal activity of these cells [49]. Data obtained *in vivo* in patients and in NHP have confirmed the absence of massive inflammatory responses during LF, by demonstrating a lack of substantial proinflammatory cytokine production despite the massive infiltration of most tissues and organs with MP and neutrophils (Table 1) [22,25,26,30,32,50]. By contrast, the MOPV infection of MP leads to cell activation, with an upregulation of surface molecules, such as CD86, CD80 and CD54, and the production of considerable amounts of IFNβ, α and λ [44,45]. However, MOPV infection does not result in the release of substantial amounts of inflammatory cytokines by MP [39,44,45]. Similarly, but to a lesser extent, MOPV infection induces a modest activation of DC, with the synthesis of mRNA encoding type I IFN [44,45]. Such a correlation between a lack of pathogenicity and an ability to activate MP has also been observed with variants of the New-World Pichinde arenavirus [51]. These observations suggest that, in arenaviruses, a lack of pathogenicity would be associated with APC activation. The type I IFN response seems to be a key element in the difference in pathogenicity between LASV and MOPV. Indeed, LASV is a poor inducer of type I IFN. Both Lassa and Mopeia viruses are equally sensitive to the antiviral properties of type I IFN, but only MOPV-infected APC can produce these cytokines [44,45,52]. The lack of type I IFN production by LASV-infected APC is probably a key element in the pathogenesis and immunosuppression observed in severe disease, as these cytokines are involved not only in the initial control of viral spread, but also in the induction of adaptive immunity [53]. Consistently, the early release of type I IFN has been observed in cynomolgus monkeys surviving severe LASV infection, whereas the production of this cytokine was detected only at terminal stages in monkeys that died [25]. In these monkeys, the high levels of IFNα circulating in the bloodstream in the last few days before death are probably unable to control the massive viral replication observed at this point in the course of the disease and may, instead, contribute to pathogenesis. Indeed, type I IFN are known to have both beneficial and detrimental effects. Type I IFN have been implicated in the transient lymphopenia and structural changes to lymphoid organs [54,55] observed during the LCMV infection of mice, and they play a crucial role in reducing platelet counts and in platelet dysfunction [56]. Additional experiments in NHP will be required to determine the sensitivity of LASV to type I IFN *in vivo*, to clarify their role in the pathogenesis of LF and to determine whether and why the innate responses induced differ as a function of outcome. Moreover, it would be important to evaluate the involvement of IFN response in the outcome of LASV infection in its natural rodent host.

### 3.3. Inhibition of Innate Immunity by LASV NP

The arenavirus NP has recently been implicated in the defective production of type I IFN in response to LASV infection. Indeed, the NP of most arenaviruses, with the exception of the Tacaribe virus, inhibit IRF3 activation and nuclear translocation and type I IFN production [57,58]. These properties are dependent on the presence, in the C-terminal part of the protein, of a dsRNA-specific 3’ to 5’ exonuclease related to the enzymes of the DEDDh family [59,60]. By digesting dsRNA, NP prevents its sensing by RIG-I and MDA-5 helicases, which have been shown to recognize arenavirus RNA [61,62], and subsequent activation of the type I IFN response [63]. The amino-acid residues required for this activity have been identified, and mutations of the corresponding nucleotides abolish the anti-IFN activity of NP [64]. However, the difference in pathogenicity between LASV and MOPV cannot be due to this property alone, as the MOPV NP also contains the DEDDh motif [64] and probably retains some ability to inhibit IFN, albeit less strongly than the LASV NP. This is suggested by the observation that recombinant LASV containing mutations in this region induce type I IFN much more strongly than MOPV [65]. 

The arenavirus NP also seems to have another string to its bow, as it inhibits IRF3 activation, as demonstrated by the recent description of its binding to the IκB kinase-related kinase IKKε, preventing autocatalytic activity and IRF3 phosphorylation [66]. The ability of NP to sequester IKKε in an inactive form, preventing the induction of innate immunity, is probably important for the virulence of LASV. It remains unclear whether the MOPV NP also has this ability. Finally, the arenavirus NP has also recently been reported to prevent the nuclear translocation and transcriptional activity of NFκB [67], consistent with the lack of APC activation and inflammatory cytokine production observed after LASV infection. Thus, like many other viruses, arenaviruses have developed efficient strategies for preventing the induction of innate immunity. Furthermore, as the NP of all tested arenaviruses are able to inhibit type I IFN response and because the only exception is the NP of Tacaribe virus, which is not a rodent-borne virus [57], it would be interesting to determine the consequences of this inhibition in the interaction between LASV and its natural rodent host.

## 4. Humoral Responses During Lassa Fever

Despite the strong antibody (Ab) responses observed in most LASV-infected patients and in NHP, no evidence of a direct role for humoral responses in the control of LF has been reported. LASV-specific IgM and IgG are rapidly produced in large amounts in patients and NHP, regardless of outcome, and are therefore not correlated with survival or with the disappearance of viremia [23,25,28,33]. The IgG has broad specificity, with Ab directed against at least NP, GP1, GP2, and Z [25,68]. However, LASV infection does not result in the induction of significant amounts of neutralizing Ab (nAb), such antibodies being detected only after recovery and, even then, only in very small amounts [25,69]. This observation contrasts with the strong induction of nAb described with New World arenaviruses and the efficiency of passive transfer reported for these viruses [70]. The lack of nAb induction during LASV infection seems to be intrinsic to GP and independent of the immunogenicity of the viral backbone [71]. Passive transfer experiments in humans and NHP infected with LASV have generated conflicting results and the efficacy of such treatment is likely to depend on the early administration of Ab with a high neutralization index [72,73,74]. In addition, immunization studies in NHP have shown that the induction of a humoral response is not sufficient to protect against a lethal LASV challenge [75,76]. Thus, although Ab seem to be unable to mediate the control of LASV replication directly, the humoral response may play an indirect role in protection. Further studies are required to determine the role of Ab during the course of the disease.

## 5. NK-Cell Responses During LASV Infection

NK cells were initially described as lymphocytes from the innate immune compartment, with cytolytic activity against tumor cells. They are also located at the crossroads between the innate and adaptive immune responses, because they secrete cytokines, such as IFN-γ, which plays an important role in regulating T-cell differentiation and functions. NK cell activation is controlled by a balance between inhibitory and activating signals from target cells [77]. The dialog between NK cells and accessory cells, such as APC, potentiates NK cell functions and the overall immune response. NK cells are involved in various viral infections. Recent studies have shown that NK cells are activated by LASV-infected MP but that they neither secrete IFN-γ nor kill the infected cells and control the infection. Similar observations have been reported for LCMV infection and for the stimulation of NK cells by MOPV-infected MP. NK cell responses during LASV infection in humans have been little studied, whereas these responses have been extensively analyzed during LCMV infections in mice [78,79]. Thus, studies of LCMV infection can provide tools to help us understand the NK cell-mediated mechanisms involved in the immune responses triggered during LASV infection.

It has recently been shown that, following their stimulation with LASV-infected MP, NK cells acquire an enhanced cytolytic potential *in vitro*, with increases in the expression of activating receptor NKp30 and granzyme B and the killing of K562 cells lacking MHC-I [80]. Moreover, the increase in TRAIL mRNA synthesis in NK/MP cocultures has been shown to be correlated with the increase in the cytolytic capacity of NK cells. NK cell-mediated cytotoxicity requires type I IFN during LCMV infection [78]. Similarly, it has been shown that type I IFN, which are produced by LASV-infected MP, are responsible for NK cell activation and the modulation of NKp30 expression, even at low levels [80]. However, infected APC remain resistant to NK cell-mediated lysis. NK cells do not control LCMV infection despite high levels of NK cell cytotoxicity [81], whereas cytolytic activity seems to play an important role in controlling Pichinde virus infection in mice [82]. LASV-infected APC express constant HLA class I molecules, which bind to inhibitory KIRs, and the expression of the activating NKG2D ligands, MIC-A/B, is also unaffected [20,80]. Negative signals, at least those provided by MHC class I, probably account for the inhibition of NK cell cytotoxicity to LASV-infected cells. This mechanism strongly resembles a game of viral “hide and seek,” rendering the infected cells insensitive to NK cell-mediated lysis.

IFN-γ is a key cytokine in the initiation and regulation of adaptive immune responses. It is directly involved in controlling the replication of many viruses. However, we and others have clearly demonstrated that IFN-γ is not induced during LASV infection. It is not detected in LASV-infected patients and NHP and its mRNA is produced, but not translated, in NK cells *in vitro*, following stimulation with LASV-infected APCs [25,80]. Moreover, IFN-γ does not control LASV replication in APCs and other cells [45,52]. No IFN-γ is induced after the *in vitro* MOPV infection of APCs either and this seems to be a common feature of LCMV infection in immunocompetent mice [83,84]. Some studies have focused on the reasons for this absence of IFN-γ secretion during LASV infection. IL-12, secreted by APC, is a well known inducer of T cell- and NK cell-mediated IFN-γ production. Several studies have shown that IL-12 is induced neither *in vitro*, following the infection of human APCs with LASV or MOPV, nor during the infection of mice with LCMV [78]. These observations suggest that the absence of NK cell-mediated IFN-γ production during LASV infection may be partly due to the lack of IL-12 secretion by LASV-infected APCs. Moreover, it has been shown that high levels of type I IFN inhibit IL-12 secretion by accessory cells and, thus, IL-12-mediated IFN-γ production by NK cells [85]. This may occur despite the very low levels detected during LASV infection.

A transient depletion of circulating NK cells and of other lymphocyte populations has been observed in LASV-infected NHP [25], suggesting that NK cells can be recruited to other tissues or depleted. This transient lymphopenia in blood is also observed during the LCMV infection of macaques [86], and can be accounted for by marginalization in the periphery and cell death. For example, NK cells are recruited to the liver during LCMV infection in mice [87,88]. NK cells express certain chemokine receptors, such as CXCR3, and they can migrate in response to chemotactic signals. CXCR3 is the receptor of CXCL9 (Mig), CXCL10 (IP-10) and CXCL11 (I-TAC), which are expressed in large numbers on LASV-infected NHP [25] and* in vitro* in humans (Pannetier *et al.*, in preparation) [80]. CXCR3 is upregulated at the surface of NK cells, where it directly senses LASV via PRRs, whereas it is downregulated when NK cells are stimulated by LASV-infected MP. We propose a model according to which CXCR3 ligands secreted by LASV-infected APC in blood vessels induce rapid desensitization and the internalization of CXCR3 at the cell surface of NK cells, resulting in the relocalization of these cells to peripheral organs. NK cells may reach secondary lymphoid organs, where they elicit immune responses, and the liver, where they mediate or regulate the hepatic inflammation occurring during LF.

NK cells have been shown to proliferate moderately in response to LASV-infected MP. The importance of NK cell proliferation during viral infections *in vivo* remains unknown. It has been suggested that the increase in NK cell populations participates in the development of NK cell memory [89]. The role of NK cells in controlling most viral infections in humans remains a matter of debate, as humans lacking functional NK cell responses do not seem to be particularly susceptibility to viral infections [78,79]. NK cells do not appear to be crucial determinants during LASV infection, as in LCMV infections. However, this requires confirmation *in vivo*, in infected patients or NHP. Infected APCs express MHC class I molecules and, via Ag processing, are susceptible to cytotoxic T cell-mediated lysis but not NK cell killing. It has recently been shown that NK cells can downregulate T cell-mediated immunity in LCMV infections of mice [90,91]. There is no evidence for the NK cell-mediated lysis of LASV-specific cytotoxic T cells, but such a mechanism may occur during LASV infection, with NK cells being responsible for the immunopathogenesis occurring during LF.

## 6. T-Cell Responses and the Control of LASV Infection

Both *in vitro* studies in human models and NHP experiments have suggested that T cells play a crucial role in the outcome of LF. Severe LASV infection seems to be associated with defective T-cell responses, whereas the effective control of LF seems to be mediated by robust and efficient responses. 

### 6.1. Defective T-Cell Immunity and Fatal Lassa Fever

Severe LF seems to be associated with defective T-cell responses. In NHP models, a general depression of T-cell responses to several mitogens has been observed in animals with fatal infections [28]. In addition to the transient lymphopenia occurring during acute disease, lymphoid depletion has also been described in the spleen and lymph nodes of NHP and humans with severe LF, together with the destruction of secondary lymphoid organ architecture [24,26]. Fatal LASV infection of cynomolgus monkeys has been associated with a lack of T-cell activation in peripheral blood and a lack of T cell-derived cytokines [25]. Similar results were reported in a study evaluating a vaccine candidate in NHP. In non immunized animals, no T-cell response was observed after a lethal challenge with LASV [92]. Similarly, LASV-infected human DC fail to activate CD4^+^ and CD8^+^ T cells in an *in vitro* model of the induction of the primary LASV-specific T-cell response [93] and in a mixed lymphocyte reaction assay [21]. It remains unclear whether the lack of induction of a T-cell response results from an active suppression of DC immunogenicity, the absence of DC activation/maturation after LASV infection or changes in the structure of lymphoid organs. Indeed, LASV-infected DC that have been matured with TNFα and IL-1β remain unable to induce efficient T-cell responses *in vitro* [93]. In addition, there may be immunopathogenic events linked to T-cell responses. Indeed, it was recently suggested that T cells may be involved in deleterious innate inflammatory reactions and pathogenesis in mice expressing humanized MHC class I [94]. In this model, interactions between infected monocytes/MP and T cells could lead to the overstimulation of MP and an exacerbation of inflammatory responses, resulting in disruption of the splenic white and red pulp compartments, a loss of the marginal zone MP layer, and severe hepatic and pulmonary damage. These data are consistent with the well-known role of T cells in the pathogenesis of the closely related LCMV [95,96]. However, further investigations are required in more relevant animal models, such as NHP, to confirm that these events are likely occur during severe LF in humans.

### 6.2. The Control of LASV Infection Is Associated with the Induction of T-Cell Responses

There is increasing evidence to suggest that T-cell responses play a crucial role in the control of LASV infection. Indeed, strong memory CD4^+^ T-cell responses directed against LASV NP and GP have been detected in LASV-seropositive healthy individuals living in zones in which LF is endemic, suggesting that mild and/or asymptomatic infections are associated with the activation of CD4^+^ T cells [97,98]. Moreover, nonfatal LASV infection in humans is associated with high serum concentrations of IL-8 and CXCL-10, two chemokines involved in the attraction and activation of T cells [99,100], whereas the concentrations of these mediators remain low in fatal cases [50]. In cynomolgus monkeys, the control of acute LF has been correlated with the circulation of activated CD4^+^ and CD8^+^ T cells six to nine days after LASV infection [25]. In these animals, a large increase in the total number of circulating T lymphocytes has also been observed, from nine days after infection. In addition, survival has been correlated with the ability of PBMC to proliferate *in vitro* in response to LASV Ag [25]. Further evidence has been provided by vaccine studies in NHP, in which protection against a lethal LASV challenge has been shown to be associated with the induction of T-cell immunity [75,92]. It has recently been shown that MOPV-infected DC induce strong and efficient specific T cells in an *in vitro* human model of the induction of primary T-cell responses, whereas LASV-infected DC induce only delayed and weak responses devoid of effective function [93]. The CD4^+^ and CD8^+^ T cells stimulated by MOPV-infected DC proliferate strongly, acquire activation and memory phenotypes and differentiate into cytotoxic T cells able to control viral infection in DC. In this model, the different T-cell responses probably result from differences in the activation of infected DC. Indeed, by contrast to the results obtained for LASV-infected DC, the coculture of MOPV-infected DC and T cells is accompanied by the strong release of type I IFN, IL-12 and CXCL-10, probably favoring T-cell activation [101,102,103]. Consistently, T cells cluster massively around MOPV-infected DC [93]. The more robust synthesis of type I IFN and IL-12 by MOPV-infected DC in the presence of T cells [93] than by infected DC alone [44] suggests cross-talk between the two populations leading to reciprocal activation and the differentiation/maturation of DC and T cells. MOPV is a nonpathogenic virus closely related to LASV that can even immunize NHP against LF. This virus is used to model non fatal LF. Thus, the T-cell responses induced by MOPV-infected DC may *de facto* reflect the immune responses induced in patients surviving acute LF and/or in individuals experiencing asymptomatic LASV infection. The main viral Ag recognized by activated T cells are probably NP and GP, as suggested by studies in humans [97,98], vaccine assays in NHP [75,76,92,104], and the prediction of potential epitopes [105,106,107]. However, the mechanisms leading to the induction of innate immunity followed by early and effective T-cell responses in survivors or to defective immune responses and fatal outcome during the course of LF remain unclear and should be investigated further. There are several possible hypotheses, including differences in inoculum size [25], different routes of infection [96,108], different cell populations targeted early in infection [109], different genetic backgrounds (MHC) and preexisting homologous or heterologous immunity [110]. These results indicate that T cells are essential for the control of LF and that a vaccine able to induce T cells specific for LASV GP, and possibly for NP, would probably be effective.

**Table 1:** Immunological features of Lassa fever in nonhuman primates as a function of outcome

Immunological parameters	Nonfatal LF	Fatal outcome	References
Inflammatory responses	High number of CD80^+^ circulating monocytes	Low number of CD80^+^ circulating monocytes	[22,25]
	Early and transient release of IFNα	Late release of IFNα	
	Inflammatory cytokines: not detected (ND)	Inflammatory cytokines: ND, except for IL-6 (late)	
	CXCL-10 and 11 mRNA	CXCL-10 and 11 mRNA	
	MCP-1 ?, eotaxin ?	MCP-1, eotaxin	
Antibodies	High levels of IgM/IgG	High levels of IgM/IgG	[25,28]
	No nAb	No nAb	
NK cells	Transient depletion from the circulation	Profound depletion	[25]
T-cell responses	T cell-derived cytokines: ND	T cell-derived cytokines: ND	[22,25]
	Transient lymphopenia followed by lymphocytosis	Marked lymphopenia	
	Early and robust activation of CD4^+^ and CD8^+^ T cells	Weak and late activation of CD4^+^ and CD8^+^ T cells	
	*In vitro* proliferation of T cells in response to LASV	No proliferation in response to LASV	

## 7. Conclusions

**Figure 1:**
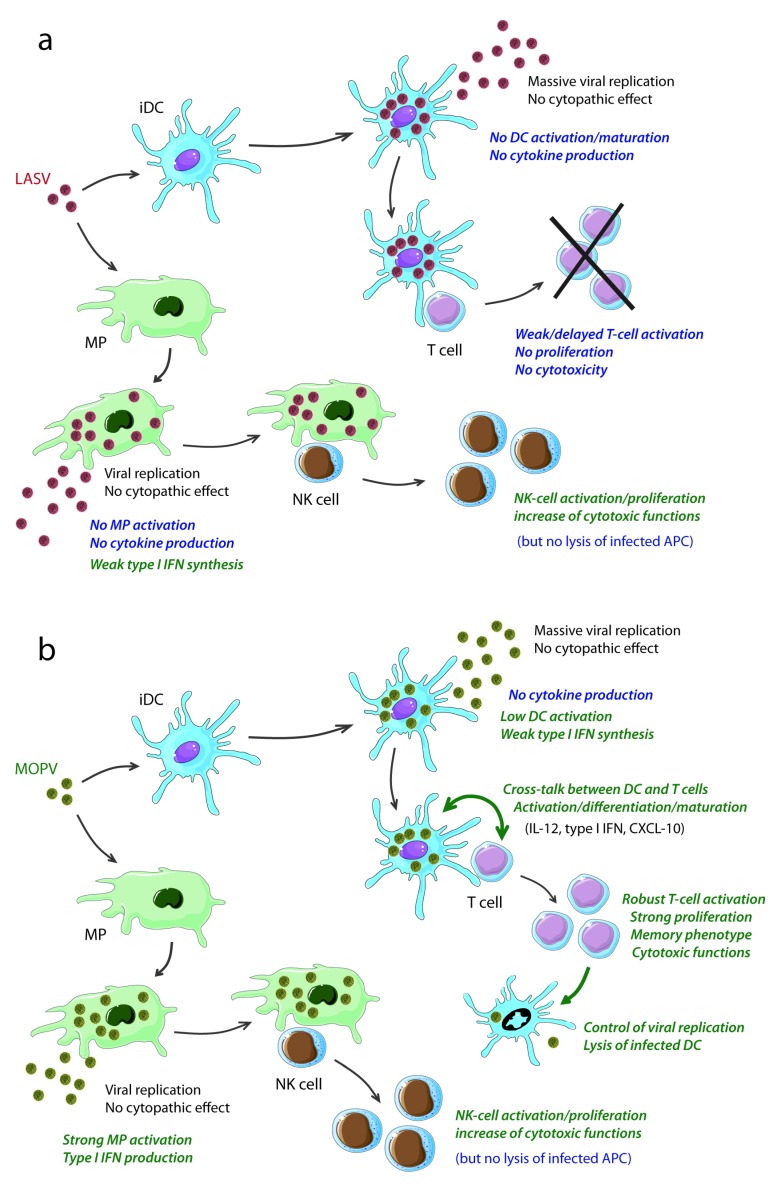
Comparison of the responses induced *in vitro *by LASV (a) and MOPV (b) in human cells (adapted from references [20,21,39,44,45,80,93])

The acquisition of knowledge about the immune responses induced during severe LF or involved in the control of acute infection has long been hampered by the need to handle LASV in BSL4 facilities, the remote location of the zones in which LF is endemic and the lack of access to patients and a relevant rodent model for studying the disease. Recently, both immunological investigation in NHP models and *in vitro* studies in human immune cells have led to major advances in our understanding of the complex interactions of LASV with the innate immune system and the responses involved in controlling viral replication. These results have provided important clues to the pathogenesis of severe disease and have highlighted the essential role of T cells in the control of LF (Figure 1), opening up new possibilities for the treatment and prophylaxis of this disease. However, the complete sequence of events leading to catastrophic illness and death and the mechanisms responsible for the control of acute infection in patients and for the ability of individuals to eliminate LASV before symptoms appear remain unclear and require further investigation.
